# Family Socioeconomic Status and Parental Involvement in Chinese Parents of Children with Autism Spectrum Disorder: A Moderated Mediation Model

**DOI:** 10.3390/healthcare11091281

**Published:** 2023-04-29

**Authors:** Tingrui Yan, Yujia Hou, Luyao Liang

**Affiliations:** 1 Special Education Department, Faculty of Education, East China Normal University, Shanghai 200026, China; tryan@ed.ecnu.edu.cn; 2 Shanghai Institute of Early Childhood Education, Shanghai Normal University, Shanghai 200234, China; 3 Macquarie School of Education, Macquarie University, Sydney 2122, Australia; luyao.liang@hdr.mq.edu.au

**Keywords:** parental involvement, family SES, children with ASD, parenting stress, China

## Abstract

Parental involvement benefits children with autism spectrum disorder (ASD) in multiple developmental areas. We conducted the present study to examine the role of parenting stress and ASD symptom severity in the relationship between family socioeconomic status (SES) and parental involvement. A total of 165 Chinese parents of children with ASD participated in this study. Mediation analyses indicated that family SES was positively related to parental involvement; parenting stress partially mediated the relationship between family SES and parental involvement. The analyses also found that ASD symptom severity moderated the influence of parenting stress on parental involvement. Specifically, the decreased parenting stress improved parental involvement when ASD symptom severity was low. The findings enhanced our understanding of the mechanism underlying the relationship between family SES and parental involvement among parents facing considerable child-rearing challenges. Implications for devising evidenced-based interventions to promote parental involvement for low SES children with ASD are discussed.

## 1. Introduction

Autism spectrum disorder (ASD) is characterized by an individual’s deficits in social communication and interaction, accompanied by repetitive and stereotyped behaviors and a restricted scope of interests [1,2]. The prevalence of ASD among Chinese children over the past two decades has attracted substantial attention from practitioners, researchers, and policymakers. Although there is no official report on the exact number of children with ASD, it is estimated that the ratio of children being diagnosed with various autistic symptoms in China has reached approximately 1 per 100 [3]; given the immense population of China, this ratio translates into some 13 million children across the country. Education and care for children with ASD have become a pressing social issue in contemporary Chinese society.

Chinese parents attach great importance to children’s education and academic success; therefore, they are usually actively involved in children’s learning; parents of children with ASD are no exception [4,5]. Parental involvement refers to a series of parental practices to promote children’s learning and development at home and school, including parents’ educational beliefs, academic expectations, and various forms of parenting behaviors [6]. Parental involvement in education plays an important role in the development of children with ASD. Extensive research has suggested that children with ASD benefit from parental involvement in various developmental areas, including improved social skill acquisition, maintenance, and generalization across school, home, and community settings [1,7] and increased opportunities for social, cognitive, and language development [8]. Furthermore, since children with ASD have difficulties communicating with their parents about their learning in schools, educational involvement is necessary and beneficial for parents to acquire information and join hands with educators to coordinate the best possible services for their children. In addition, parental involvement provides teachers with critical information about family values, routines, and children’s strengths and weaknesses, based on which educators and specialists can design more targeted interventions for children with ASD [9]. Nevertheless, several studies have demonstrated that low family socioeconomic status (SES) is a salient risk factor that undermines the effect of parental involvement [10,11]. This gives rise to numerous challenges for the large number of Chinese parents who not only face the difficulty of raising children with ASD, but also live in low socioeconomic conditions. In comparison to parents with higher SES, parents of lower SES show disadvantages in the frequency and quality of their involvement in their children’s education [12]. For parents raising children with ASD, prior studies have found that family SES, such as parents’ low education level and household income, can constrain their ability to make decisions to meet the children’s educational needs [13,14].

According to Belsky’s (1984) process model of parenting [15], parenting behaviors are shaped by family contextual sources, parental psychological traits, and children’s characteristics. In addition to family SES, two other influencing factors of parental involvement, namely, parenting stress and ASD symptom severity, have received extensive attention from researchers [5,8]. According to Eccles and Harold’s (1996) parental involvement model [16], the association between family SES and parental involvement reflects the processes in which the distal contextual factors affect parental involvement via the proximal factors internal to them. Therefore, family SES, as a family contextual source, may influence parental involvement through parenting stress and children’s ASD symptom severity. However, to date, little is known about the relationship between family SES and parental involvement and the potential role of parenting stress and ASD symptom severity in the Chinese context. We set out to examine this matter in this study.

### 1.1. Family Socioeconomic Status and Parental Involvement

It has been shown by prior research that family SES is a strong predictor of parental involvement [17,18]. It reflects the degree to which a family possesses, receives, and manages the available resources, including wealth, power, social status, etc.; it is usually measured by parents’ educational and income level [19]. Previous research has found that parents with higher levels of income and education are more likely to participate in children’s education, indicating that the family’s socioeconomic resource plays an important role in increasing parental involvement [20]. In general, upper-middle-SES parents feel more comfortable communicating with teachers when participating in school activities than lower-SES parents do [21]. Lee and Bowen (2006) found that compared to parents with higher educational degrees, parents with lower educational levels showed considerably less attendance in the activities or meetings organized by the school; they also tended to talk less about educational issues with their children and had lower expectations for their children’s academic achievement [22].

For parents of children with ASD, research evidence has suggested that family SES is an important contextual factor affecting parental involvement [23]. Norbury and Sparks (2013) found that parents with low SES faced the priority of maintaining a residence and providing for the family; consequently, they tended to spend less time on educating their children with ASD compared to those who had no such family economic pressure to attend to [24]. Similarly, Benson et al. (2008) found that family SES exerted a significant positive effect on maternal home-based educational involvement [13]. However, despite the established link between family SES and parental involvement, it is unlikely and infeasible for all family-based interventions to include direct financial support to raise family SES. Thus, empirical research needs to target potentially amenable factors that may improve parental involvement among those who raise children with ASD. In this vein, we conducted this research to examine the internal mechanism by which family SES affects parental involvement among a group of families facing substantial child-rearing difficulties.

### 1.2. Mediation Effect of Parenting Stress between Family SES and Parental Involvement

Parenting stress is conceptualized as a stressful experience in which parents fulfill their child-rearing roles while undergoing various negative psychological episodes such as anxiety, frustration, and self-blame [25,26]. In particular, parents raising children with ASD are more likely to experience substantial psychological, financial, and physical burdens, given the severity of their children’s disabilities [27]. Previous studies have reported that compared to parents of children with other developmental disorders, such as Down’s syndrome or developmental delay, parents of children with ASD typically report higher levels of parenting stress and depression [26,28]. Among non-ASD groups, the links between parenting stress and parental involvement have also been revealed, with recent evidence showing the negative effects of cumulative stress on parental involvement [29]. Consistent with this notion, Yao and Liu (2018) reported that stressful events throughout the lifespan could produce a spillover effect in which the negative emotions of family members transferred from one situation to another under long-term high pressure, which reduced the motivation, frequency, duration, and quality of parental participation in children’s education [30]. According to Hoover-Dempsey et al.’s (2005) family involvement model [31], parenting stress is identified as an adverse psychological factor hindering parents’ involvement in children’s education. Other research has also indicated that the high level of stress associated with raising a child with severe behavioral challenges could lead to parents’ low level of self-efficacy, which in turn, discourages them from actively engaging in children’s educational activities [13,32]. Similarly, the parenting stress of raising a child with ASD has been identified as a factor associated with the negative long-term outcome of parental involvement, such as less parent–child interaction and low frequency of home–school communication [8,33].

Prior research has demonstrated the association between family SES and parenting stress [34,35,36]. Some researchers have found that mothers from low-SES households are more likely to undergo uncontrollable negative life events and stressful experiences, which significantly increase the risk of developing mental health problems, including parenting stress [37,38]. Moreover, the combined effect of low SES and parenting stress may result in family dysfunctions that can further worsen the quality of parent–child interaction and parental involvement [39]. According to the family stress model [40], stressors such as socioeconomic strains (i.e., low family socioeconomic status) can result in psychological distresses, including depression and anxiety, thereby leading to less involved parenting. Consistent with this model, Emmen et al.’s (2013) research demonstrated that the relation between family SES and positive parenting was partially mediated by general maternal psychological stress and maternal acculturation stress [35]. Likewise, it is plausible that high family SES can provide social support and proximal resources that help parents cope with parenting stress, thereby improving parental involvement. In contrast, low family SES may give rise to parenting stress, which further undermines parental involvement. However, there is a paucity of empirical research examining this mediation mechanism among parents of children with ASD; we conducted the present study to fill this knowledge gap.

### 1.3. Moderation Effect of ASD Symptom Severity

Research demonstrates that children found on the two ends of the autism spectrum show different developmental trajectories and outcomes in the domains of cognition, emotion, and behavior [41]. Therefore, the severity of children’s autism symptoms should be accounted for when studying the family environment and the child–caretaker relationship. Levinson et al.’s (2021) recent study showed that parents who raised children with severe ASD symptoms were likely to develop negative beliefs regarding the education of their children, which translated into their decreased involvement in a variety of educational activities [42]. This finding corroborates that of Benson et al.’s (2008) research [13], which shows that the children’s display of severe behavioral difficulties (e.g., lack of functional language and inability to maintain ongoing social interaction with caregivers) resulted in less parental involvement. In contrast, more parental involvement was observed when children with ASD demonstrated abilities to follow directions and maintain focus on the given tasks.

The current literature has also shown that the severity of children’s ASD symptoms interacts with family SES and parenting stress in the production of influence on parental involvement. For example, McNeal (2001) found that the parental involvement of low-SES parents was significantly lower than that of high-SES parents when children showed only mild behavioral problems [43]. However, such a significant difference was not observed when children displayed severe behavioral problems. Semke et al. (2010) found that reducing parenting stress for parents of children with severe disruptive behaviors alone failed to significantly increase their parental involvement [32]; this is due to the parents’ low self-efficacy in their ability to help children achieve educational pursuits. In addition, mothers who experienced high stress levels from taking care of children with ASD rated their children’s behavioral problems as more severe than those who experienced less parenting stress [44]. Based on these research studies, we speculated that children’s ASD symptom severity might moderate the direct or indirect relationship between family SES and parental involvement. However, there is a dearth of empirical research evidence clarifying the potential moderation role of ASD symptoms. We set out to test the speculation among a group of parents raising children with ASD.

### 1.4. Parental Involvement in Children with ASD in China

Conducting culturally responsive research to understand parenting characteristics in different cultures serves as an important prerequisite for creating and improving learning opportunities for children with ASD. However, most studies focusing on parental involvement and its impact on children with ASD relied on samples drawn from Western societies [45], leaving parental involvement in non-Western cultures much less understood. Therefore, it is necessary to examine how family SES, parenting stress, and children’s developmental characteristics influence parental involvement in Chinese society, where parents typically hold high expectations for their children and are highly responsive to their children’s educational needs [46].

In China, the national education policy and the traditional Confucius beliefs emphasize the significance of parental involvement in children’s education. In 2016, the Chinese government issued the Five-year Plan on Guiding and Promoting Family Education (2016–2020) [47]. This government document rigorously promotes parental involvement as a critical parenting aspect that gives rise to children’s academic success and all-round development. Such governmental attitude reflects the deeply rooted Confucius teaching that parents are responsible for participating in their children’s education to help them pursue academic excellence, which would ultimately lead to a happy and successful life for children and bring honor to the whole family [48].

Despite such a socio-cultural background, prior studies have found that Chinese parents of children with ASD tend to be less involved in their children’s educational activities. For example, Xiong and Sun’s (2014) study found that parents of children with ASD demonstrated little or no involvement in their children’s individualized education programs (IEP) or individual family service plans (IFSP) [49]. The lack of social support and relevant social resources may have restricted these parents’ capability to participate in their children’s educational activities [50]. It might be even more so for parents from low-SES households, who are forced to devote all their energy and efforts to supporting the family. To further complicate the situation, parents raising a child with a developmental disorder are more prone to be discriminated against in a society where the public knows little about and holds biased opinions towards the disabled [51]. This could lead to parents’ elevated sense of guilt and demotivate them from engaging in their children’s education. Although Chinese parents with typically developing (TD) children are usually actively involved in their children’s education regardless of the family SES, it may not be the case for parents raising children with ASD. However, the impact mechanism underlying the relationship between family SES and parental involvement has received little research attention in the Chinese context. Filling this research gap would address this knowledge gap from a cross-cultural perspective and provide insights for policymakers to design more targeted intervention programs to increase parental involvement of children with ASD.

### 1.5. The Present Study

Little empirical research has been conducted to investigate the association between family SES and parental involvement while accounting for the potential role of parenting stress and ASD symptom severity in non-Western societies. This warrants culturally responsive research to tap into specific parenting matters in a different socio-cultural context. Therefore, we conducted this study to investigate the internal mechanism that underpins the relationship between family SES and parental involvement in contemporary Chinese society. The mediator and moderator are two different variables, in which the mediator explains the process or mechanism through which the independent variable causes the dependent variable, and the moderator influences the strength or direction of the relationship between the independent variable and the dependent variable [52]. We hypothesized:

**H1.**  *Higher family SES is associated with higher parental involvement;*

**H2.**  *Parenting stress mediates the relationship between family SES and parental involvement;*

**H3.**  *ASD symptom severity moderates the relationship between family SES and parental involvement, with the relationship being stronger for children with lower ASD symptom severity;*

**H4.**  *ASD symptom severity moderates the mediating effect of parenting stress in the relationship between family SES and parental involvement, with the mediating effect of parenting stress being stronger for children with lower ASD symptom severity.*

**Figure 1:**
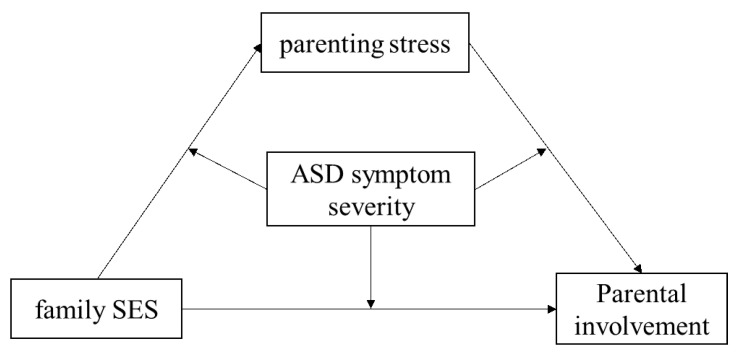
Hypothesized model.

In sum, we proposed a moderated mediation model in which parenting stress mediates the association between family SES and parental involvement, with ASD symptom severity moderating the direct effect of family SES on parental involvement while also moderating the mediating effect of parenting stress (see Figure 1).

## 2. Materials and Methods

### 2.1. Participants and Sample

Participants of this study were all parents of children diagnosed with ASD. They were recruited by convenience sampling from five cities in China: Beijing, Qingdao, Xiamen, Weifang, and Zhengzhou. First, the researchers established the following eligibility criteria for selecting and including participants: (a) parents served as the children’s primary caregivers at home; (b) the children were diagnosed with ASD by certified medical institutions or health professionals, as per the Chinese Classification of Mental Disorders (3rd ed); and (c) parents were capable of completing the questionnaire independently. Second, the researchers contacted the principals of ten kindergartens and seven public schools that included children with ASD in their programs, and briefed the principals about the eligibility criteria and the purpose of the research. After gaining the principals’ written consent, the researchers invited them to introduce the research to the target parents in their respective educational settings. In total, 186 parents volunteered to participate in this research project. Among them, 178 were eligible according to the inclusion criteria abovementioned. Third, the researchers sent these parents a package containing an information letter and the consent forms. Fourth, after receiving these parents’ written consent, the researchers sent them the questionnaire and invited either the mother or father of the child(ren) to complete them. Fifth, after receiving the returned questionnaires, the researchers conducted an initial screening, in which 13 were excluded due to incomplete data, leaving 165 Chinese parents raising children with ASD included in the final sample. Among them were 137 mothers and 28 fathers. Parents’ ages ranged from 27 to 41 (M = 35.33, SD = 4.53). Children’s ages ranged from 2 to 12 (M = 5.42, SD = 1.83), with most of them being males (83.0%). The demographic characteristics of the participants are illustrated in Table 1.

### 2.2. Measures

#### 2.2.1. Family Socioeconomic Status (SES)

Consistent with Emmen et al. (2013) [35], the family SES was measured by a composite variable based on the parents’ educational levels and their household income. Parents rated their highest educational degree on a 5-point Likert-type scale: 1 (below high school), 2 (high school degree), 3 (junior college degree), 4 (bachelor’s degree), and 5 (graduate degree or above). In terms of family income, parents reported the family monthly income on a 4-point Likert-type scale, with 1, 2, 3, and 4 representing the income level of below ¥5000, ¥5000–10,000, ¥10,000–15,000, and above ¥15,000, respectively. In line with previous studies [35,53], we converted the scores of family income and the highest parental education level into standard scores, using their sum as a proxy for family SES.

#### 2.2.2. Parenting Stress Index Short Form (PSI-SF)

We adopted the Chinese version of the PSI-SF [25] to measure the parents’ perceived parenting stress. The PSI-SF comprises 36 items divided into three subscales: parental distress, parent–child dysfunctional interaction, and difficult children. This scale has been widely applied to the ASD population [54]. Parents of the children rated their level of agreement with each of the statements on a 5-point Likert-type scale ranging from 1 (completely disagree) to 5 (completely agree). The item scores in three subscales were added up to create a total score, with a higher score representing more parenting stress. Prior research conducted with Chinese participants has demonstrated the high internal consistency, test–retest reliability, and validity of PSI-SF [55]. In the present study, Cronbach’s alpha for parental stress, parent–child dysfunctional interaction, and difficult children were 0.93, 0.84, and 0.72, respectively.

#### 2.2.3. Family Involvement Questionnaire-Short Form (FIQ-SF)

Parental involvement was measured using the Chinese version of the Family Involvement Questionnaire-Short Form adapted by Liu and Li (2019) based on the original Family Involvement Questionnaire-Short (FIQ-S) [56,57]. The 20-item questionnaire consists of three subscales: home–school communication, involvement at home, and involvement at school. All the items were rated by the parents using a 4-point Likert-type scale (1 = very rarely to 4 = very often). All the responses in three subscales were aggregated to produce a total score, with a higher score indicating a high level of parental involvement. Prior research has reported good internal consistency using this measurement with Chinese participants [58]. In this study, Cronbach’s alpha for home–school communication, involvement at home, and involvement at school were 0.91, 0.87, and 0.85, respectively.

#### 2.2.4. Social Responsiveness Scale (SRS)

The Chinese version of the Social Responsiveness Scale (SRS) is revised by Cen (2017) based on Constantino and Gruber’s (2005) work [59,60]. It assesses a wide array of autistic symptoms and traits displayed by children. This 65-item scale is a caregiver-reported measure consisting of five subscales: social awareness, social cognition, social communication, social motivation, and autistic mannerisms. The SRS was completed by the parents using a 4-point Likert scale (not true = 0, sometimes true = 1, often true = 2, almost always true = 3). High total score indicates high level of autistic symptoms. The scale obtained high level of internal consistency (Cronbach’s alpha = 0.93) in this study, as with other studies [61], indicating that SRS could serve as a valid measure of ASD symptom severity among Chinese children.

### 2.3. Control Variables

Previous studies have shown that parents’ genders, ages, the number of children, and children’s genders were associated with parenting stress and parental involvement [62,63]. To determine the control variables of this study, T-test, one-way analysis of variance (ANOVA), and Pearson correlation were applied to analyze the differences in parenting stress and parental involvement across such independent variables as parents’ genders, ages, children’s genders, ages, grades, and the number of children in the family. Our analyses revealed significant differences in parenting stress among children of different grades (t = −2.21, *p* < 0.05) and a positive link between parenting stress and children’s ages (r = 0.19, *p* < 0.05). In subsequent analyses, children’s ages and grades were treated as covariate variables.

### 2.4. Data Analysis

We used Statistical Package for Social Sciences (SPSS) 24.0 to perform all statistical analyses. First, the descriptive statistics, such as frequency distributions, means, and standard deviations, were analyzed to summarize the demographic characteristics. Second, the Pearson correlation was conducted to examine the relationships between family SES, parenting stress, parental involvement, and ASD symptom severity. In addition, *t*-test, ANOVA, and Pearson correlation were used to determine control variables by analyzing whether the main variables (e.g., parenting stress, parental involvement) differ across demographic variables.

Third, G*power 3.1.9.2 software developed by researchers from University of Dusseldorf in Germany was used to assess the power of the mediation and moderation analyses with the given sample. The power analysis of post hoc estimation reported that the power (1 − β) = 0.94 (f_2_ = 0.15, α = 0.05), suggesting a higher statistical power. Third, mediation analyses were executed using the PROCESS macro for SPSS (model 4) to test Hypothesis 2. This model (PROCESS model 4) yielded three serial regression models for family SES, parenting stress, and family SES * parenting stress as predictor variables. The value of the variance inflation factor for family SES and parenting stress were 1.008 and 1.042, respectively, indicating that the issue of multicollinearity did not occur in the hierarchical regression analysis [64].

PROCESS automatically generated an interaction term between the mean-centered independent variable (family SES) and the centered mediating variable (parenting stress) and analyzed the regression model, including the centered independent, the centered mediating variable, the interaction term, and covariates (children’s ages and grades). The significance of mediating effect was estimated based on the bootstrapping with 5000 samples by 95% bias-corrected confidence intervals (CI). The mediating effect would be significant if the confidence intervals did not contain 0 [65].

Fourth, a conditional process model was performed using PROCESS macro for SPSS (model 59) to test the moderated mediating effect of ASD symptom severity. This model allowed the direct and/or indirect effect of family SES (X) on parental involvement (Y) via parenting stress (M) to be moderated by ASD symptom severity (V). This conditional indirect effect implies that the association between an independent variable and a dependent variable is subject to change based on the value of one or more moderators [66]. In addition, a bootstrapping approach was adopted to test the conditional indirect effects, which generated the confidence intervals for the conditional indirect effects by repeating 5000 random samples [67]. Lastly, the unstandardized coefficients for the simple slopes were generated to decompose all significant interaction effects.

## 3. Results

### 3.1. Descriptive Statistics

Table 2 presents the results of the descriptive analyses and the correlational analyses among the variables. The Pearson product–moment correlation analysis showed that the family SES was significantly and positively correlated with parental involvement for children with ASD (r = 0.21, *p* < 0.01); meanwhile, it was significantly and negatively correlated with parenting stress (r = −0.20, *p* < 0.05). In addition, parenting stress was significantly and negatively correlated with parental involvement for children with ASD (r = −0.28, *p* < 0.01); while it was also significantly and positively correlated with children’s ASD symptom severity (r = 0.55, *p* < 0.01). However, family SES was not significantly correlated with ASD symptom severity.

### 3.2. The Mediation Effect of Parenting Stress

Based on the Pearson correlation results showing that family SES and parenting stress were significantly correlated with parental involvement for children with ASD, we further explored whether parenting stress mediated the relationship between family SES and parental involvement (see Figure 1).

We performed a series of mediating effect analyses using PROCESS for SPSS 24.0. Children’s grades and ages were included in the mediation model as control variables due to their significant relationships with the mediated variables (parenting stress). As Table 3 shows, family SES positively predicted parental involvement (B = 0.228, SE = 0.077, and *p* < 0.01), indicating the significant direct effect of family SES on parental involvement. However, family SES negatively predicted parenting stress (B = −0.190, SE = 0.077, and *p* < 0.01). Our analysis also showed that parenting stress negatively predicted parental involvement (B = −0.263, SE = 0.077, and *p* < 0.01). An indirect relationship indicated that lower family SES led to more parenting stress, which predicted less parental involvement. To test the significance of the mediating effect of parenting stress, we adopted a bias-corrected bootstrap estimation approach with 5000 samples. The results showed a significant indirect effect (95% CI = (0.004, 0.100)). In sum, these findings suggested that the relationship between family SES and parental involvement was partially mediated by parenting stress.

### 3.3. The Moderator Mediation Effect of ASD Symptom Severity

We employed the Model 59 of the SPSS PROCESS macro to test the moderated mediation model. According to Hypotheses 3 and 4, ASD symptom severity may moderate the indirect and direct effect of family SES on parental involvement. As Table 4 shows, after controlling for children’s grades and ages, the interaction between family SES and ASD symptom severity exerted no significant effect on either parenting stress (B = −0.050, SE = 0.063, and *p* > 0.05) or parental involvement (B = 0.022, SE = 0.069, and *p* > 0.05). Notably, only the interaction between parenting stress and ASD symptom severity significantly affected parental involvement (B = 0.177, SE = 0.058, and *p* < 0.01). This finding indicates that the relationship between parenting stress and parental involvement is moderated by ASD symptom severity.

**Figure 2:**
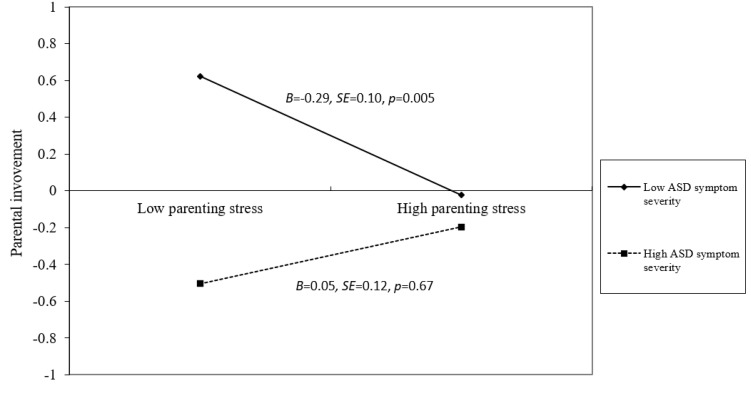
Effects of parenting stress and ASD symptom severity on parental involvement.

Subsequently, a data plot based on the guidelines developed by Aiken and West (1991) [68] visually presented the interaction effect of parenting stress and ASD symptom severity on parental involvement (Figure 2). Figure 2 shows the predicted values derived from the simple slope test. It demonstrated parents’ involvement at high (one SD above the mean) and low (one SD below the mean) parenting stress levels with high/low levels of ASD symptom severity. The results indicated that at low levels of ASD symptom severity, parents with lower parenting stress demonstrate more parental involvement after controlling for children’s grades, ages, and family SES (B = −0.29, SE = 0.10, *p* < 0.01). In contrast, when ASD symptom severity reaches a high level, parenting stress does not affect parental involvement after controlling for children’s grades, ages, and family SES (B = 0.05, SE = 0.12, *p* > 0.05).

## 4. Discussion

The present study examined the direct and indirect effects of family SES on parental involvement among Chinese parents raising children with ASD, who are especially susceptible to experiencing stress and frustration while interacting with their children [5]. Although it has been reported by previous studies that low family SES is a risk factor that could bring a negative influence on parental involvement [69], little did we know about the mechanism through which family SES affects how parents engage themselves with children’s education, particularly in families raising children with ASD. This study extended the literature by constructing a moderated mediation model to illustrate how family SES predicted parental involvement among a sample of Chinese parents.

By conducting the present study, we found that family SES positively predicted parental involvement among children with ASD. This finding corroborates with existing research evidence found in China and Western societies that shows parents with higher family SES are more involved in their children’s school-related activities and parent–child activities, and provide more resources than parents with low family SES among children with ASD [13,70]. According to the family investment theory, parents with high family SES are likely to invest more resources and time in parent–child interaction and home–school communications [40]. In contrast, research shows that many low-SES Chinese parents of children with ASD devote a substantial amount of time to make a living as well as to cover the high cost of obtaining intervention and rehabilitation for the children; therefore, these parents have much less spare time to engage in their children’s educational activities in school or at home [71]. At present, China has evolved into very different social stratification, such as state and social managers, industrial workers, enterprise managers, private business owners, professional and technical personnel, agricultural laborers, etc. [72]. In such a socio-cultural context, less parental involvement resulting from low SES is likely to result in many negative influences on the development of children with ASD, including poor academic outcomes and severe emotional or behavioral problems [73].

Our study confirmed the hypothesis that the effect of family SES on parental involvement is mediated by parenting stress among children with ASD. This finding supports a growing body of research showing the impact of family SES on parenting stress and the impact of parenting stress on parental involvement among children with ASD [74,75]. More specifically, we found that low family SES leads to higher levels of parenting stress, which in turn, reduces parental involvement in education for children with ASD. This result echoes the family stress model, which posits that low family SES (e.g., experiencing family financial difficulty) could transform into parents’ psychological stress [40]. Consequently, parents may feel frustrated and emotionally distressed, which prevents them from engaging in effective parenting practices. For low SES parents of children with ASD, the predicament of possessing limited family resources and having to spend a large share of family income on rehabilitation training inevitably leads to a high level of parenting stress [76]. These practical difficulties may render parents deeply frustrated and cause them to be less involved in the education of their children with ASD. Therefore, understanding the underlying mechanism through which family SES influences parental involvement through parenting stress can inform policymakers and program designers to develop more effective social support to assist these parents in their child rearing.

In addition, our findings partially supported Hypotheses 3 and 4, that ASD symptom severity moderated the direct and indirect relationship between family SES and parental involvement. Unexpectedly, we found that ASD symptom severity did not buffer the immediate effect of family SES on parenting stress and parental involvement. We propose that this discrepancy reflects that the effect of family SES on parents’ psychology and behavior is not influenced by children’s characteristics among families raising children with ASD. This speculation is in line with Manstead’s (2018) suggestion that socioeconomic status, to a certain extent, determines an individual’s behavior, thoughts, and feelings [77].

Lastly, our study revealed the moderating effect of ASD symptom severity on the relationship between parenting stress and parental involvement. Specifically, our analyses showed that low ASD symptom severity reduced the adverse effect of high parenting stress on parental involvement, but not when ASD symptom severity was high. Although this finding contradicts the stress-buffering hypothesis [78], it corroborates the results of Osborne and Rhodes’s (2001) research that intervention strategies targeting improving parental involvement by reducing their stress are only effective under low ASD symptom severity conditions [79]. With children exhibiting less severe ASD symptoms, such as in following directions, communicating, and sharing with parents, the parenting stress and the anxieties associated with caring for a child with ASD are reduced, thus encouraging more parental involvement. In contrast, high ASD symptom severity diminishes parents’ efforts and willingness to engage in children’s educational programs.

## 5. Conclusions

Overall, the present study makes several valuable contributions to the current literature on ASD and parental involvement. First, although prior studies have recognized the importance of family SES in parental involvement in education for children with ASD, the current study provides further insights into how family SES influences parental involvement via direct and indirect pathways, with the mediation of parenting stress and the moderation of ASD symptom severity. Second, this study lends support to Belsky’s (1984) process model of parenting that emphasizes family SES as an important family contextual factor affecting parenting practices [15], with parental psychological traits (parenting stress) and children’s characteristics (ASD symptom severity) playing additional roles in this association.

Several limitations of this study should be noted. First, this study did not examine the potential differences in mothers’ and fathers’ involvement in their children’s education; future studies should include a more gender-balanced sample to investigate whether the mechanism of family SES affecting parental involvement differs for fathers and mothers. Second, self-report data from parents in this study may lead to some measurement errors or biased responses. Despite the guarantee that their responses would remain confidential, participants may still feel reluctant to report high levels of parenting stress and the true severity of their children’s ASD symptoms. Particularly, despite special reminders in the questionnaire, the diagnosis of ASD in children was reported by their parents, which may have led to an inaccurate sampling of participants. Future research may consider using alternative data collection methods, such as a combination of parent self-reports and teacher reports. Third, the present study is correlational in nature, which does not allow causal inferences to be drawn. Thus, experimental design is warranted in future research to make more robust inferences about the causal relationship between family SES, parenting stress, ASD symptom severity, and parental involvement. Finally, the form of parental involvement and the support provided to children with ASD in this involvement may vary at different stages of education (e.g., preschool, primary school, and junior school), which were not fully examined in the current study. Although the child’s grade was included as a control variable in the mediation and moderation analysis, future research should further focus on the differences in parental involvement between different education stages.

Despite these limitations, the findings of this study have important practical implications from a clinical perspective. First, low SES parents demonstrate a high level of parenting stress, which leads to low parental involvement. Policymakers, educators, and other social welfare professionals should focus more attention on developing targeted measures to reduce parenting stress (e.g., anxiety and depression) of low SES parents who raise children with ASD. They may consider some of the approaches based on research findings that aim to optimize social support for families of children with ASD. These potential approaches include individual and family interventions, community interventions, and service-related interventions [80]. In the literature, mindfulness-based interventions, Acceptance and Commitment Therapy (ACT), and Emotionally Focused Therapy (EFT) have all been found to be effective in teaching parents to cope with negative experiences and, thus, enhancing parents’ well-being [81,82,83]. In addition, as ASD symptom severity plays a moderating role between parenting stress and parental involvement, evidence-based interventions for children with ASD, such as discrete trial teaching (DTT), parent-implemented intervention (PII), and functional behavior assessment (FBA) [84] should be adopted to reduce children’s emotional and behavioral difficulties, thereby improving parent–child relationships, and promoting parental involvement. Furthermore, schools should develop parent involvement programs specifically for low-SES families. For example, organizing workshops, holding family reading sessions, keeping communication channels open with lower-SES parents, etc., are all measures that can help guide parents’ involvement in children’s education and improve the quality of home–school partnerships.

## Figures and Tables

**Table 1:** Descriptive characteristics of the participants (N = 165).

Characteristics	Mean (SD)	N	Percentage (%)
Children’s age	5.42 (1.83)		
Children’s gender			
Male		137	83.0
Female		28	17.0
Grade			
Kindergarten		108	65.5
Primary school		57	34.5
One child or more			
Only one child		104	63.0
More than one		61	37.0
Parents’ gender			
Male		31	18.8
Female		134	81.2
Parents’ age	35.33 (4.53)		
Educational level			
Junior high school and below		12	7.2
High school diploma		26	15.8
Junior college degree		38	23.0
Bachelor		78	47.3
Master and above		11	6.7
Family income			
Below ¥5000		42	25.5
¥5000–¥10,000		70	42.4
¥10,000–¥15,000		36	21.8
Above ¥15,000		17	10.3

**Table 2:** Descriptive statistics and Pearson’s correlations among variables.

Variables	M (SD)	Range	1	2	3	4
1. Parenting stress	102.87 (21.00)	46–156	1			
2. Parental involvement	54.29 (9.66)	30–80	−0.28 **	1		
3. ASD symptom severity ^1^	160.45 (24.93)	84–217	0.55 **	−0.34 **	1	
4. Family SES ^2^	0.01 (0.99)	−2.25–2.30	−0.20 *	0.21 **	−0.07	1

* *p* < 0.05 and ** *p* < 0.01. ^1^ ASD = autism spectrum disorder. ^2^ SES = Socioeconomic Status.

**Table 3:** Regression results for the mediation model.

Outcome Variables	Predictors	B	SE	t	R^2^	F
Parental involvement	Grade	−0.139	0.083	−1.681	0.065	3.754 ***
	Child’s age	0.106	0.083	1.287		
	Family SES	0.228	0.077	2.969 **		
Parenting stress	Grade	0.050	0.082	0.603	0.073	4.247 ***
	Child’s age	0.162	0.082	1.966		
	Family SES	−0.190	0.077	−2.480 **		
Parental involvement	Grade	−0.126	0.080	−1.571	0.129	5.939 ***
	Child’s age	0.149	0.081	1.839		
	Family SES	0.178	0.076	2.353 *		
	Parenting stress	−0.263	0.077	−3.427 **		

* *p* < 0.05, ** *p* < 0.01, and *** *p* < 0.001.

**Table 4:** Regression results for the moderated mediation model.

Dependent Variables	Predictor Variables	B	SE	95%CI		t	R^2^	F
Parenting stress	Grade	−0.028	0.071	−0.168	0.112	−0.397	3.334	15.967 ***
	Child’s age	0.086	0.071	−0.054	0.226	1.220		
	Family SES	−0.154	0.065	−0.284	−0.025	−2.356 *		
	ASD symptom severity	0.517	0.067	0.386	0.649	7.775 ***		
	Family SES × ASD symptom severity	−0.050	0.063	−0.175	0.075	−0.788		
Parental involvement	Grade	−0.071	0.077	−0.224	0.081	−0.926	0.229	6.668 ***
	Child’s age	0.150	0.077	−0.003	0.303	1.942		
	Family SES	0.211	0.073	0.067	0.354	2.905 **		
	Parenting stress	−0.088	0.086	−0.259	0.082	−1.024		
	Parenting stress × ASD symptom severity	0.177	0.058	0.062	0.292	3.040 **		
	ASD symptom severity	−0.250	0.085	−0.418	−0.081	−2.929 **		
	Family SES × ASD symptom severity	0.022	0.069	−0.114	0.159	0.325		

* *p* < 0.05, ** *p* < 0.01, and *** *p* < 0.001.

## Data Availability

Data are available by contacting the corresponding or first authors.
